# Dexamethasone-cyclophosphamide pulse therapy outcomes comparing pemphigus vulgaris and pemphigus foliaceus groups in a Brazilian cohort study^☆^

**DOI:** 10.1016/j.abd.2022.11.005

**Published:** 2023-06-22

**Authors:** Ludmilla Figueiredo Fontenelle, Roberto Bueno-Filho, Sebastián Vernal, Renata Delfino, Giovanna Stefanne Lópes Barbosa, Eduardo Antonio Donadi, Ana Maria Roselino

**Affiliations:** a Department of Medical Clinics, Hospital Universitário, Universidade de São Paulo, Ribeirão Preto, SP, Brazil; b Department of Medicine, Universidade Federal do Delta do Parnaíba, Parnaíba, PI, Brazil; c Department of Medical Clinics, Universidade de São Paulo, Ribeirão Preto, SP, Brazil

**Keywords:** Corticoids, Cyclophosphamide, Pemphigus, Pulse therapy, drug

## Abstract

**Background:**

Dexamethasone-cyclophosphamide pulse (DCP) and dexamethasone pulse (DP) have been successfully used to treat pemphigus, but DCP/DP outcomes comparing pemphigus vulgaris (PV) and pemphigus foliaceus (PF) are scarce.

**Objective:**

To compare DCP/DP outcomes in a Brazilian cohort of PV and PF patients according to demographic and clinical data.

**Methods:**

Retrospective analytical cohort study, reviewing medical charts of PV and PF patients (for DCP/DP Phases I‒IV consult Pasricha et al.16‒18).

**Results:**

37 PV and 41 PF patients non responsive to usual treatments were included similarly for DCP or DP therapy. Disease duration was longer among PF before DCP/DP prescription (p < 0.001); PF required a higher number of monthly pulses to acquire remission in Phase I (median 10 and 6 pulses, respectively; p = 0.005). DCP/DP outcomes were similar in both groups: remission in 37.8% of PV and 34.1% of PF after completed DCP/DP cycles following a median of 13 months (1–56 months follow-up); failure occurred in 13.5% of PV and 14.6% of PF in Phase I; relapse in 13.5% of PV and 12.2% of PF, and dropout in 27% of PV and 24.4% of PF in Phases II to IV. Mild side effects were documented.

**Study limitations:**

The severity of PV and PF disease was not assessed by score indexes.

**Conclusions:**

PV and PF patients presented similar DCP/DP outcomes. DCP/DP should be initiated earlier in PF patients due to the longer duration of their disease in order to decrease the number of pulses and the duration of Phase I to acquire remission.

## Introduction

Pemphigus comprehends a group of autoimmune bullous diseases characterized by IgG autoantibodies production against desmogleins.^1^, ^2^ Both pemphigus vulgaris (PV) and pemphigus foliaceus (PF) clinical forms are prevalent in some Brazilian regions, whose pathogenesis has yet been extensibility studied.^3^, ^4^, ^5^, ^6^ In Southeastern Brazil, HLA susceptibility/protective alleles/haplotypes have been identified, differentiating PV from PF in terms of HLA genetic profile.^7^

The main pemphigus treatment outcome is to reach clinical remission. The standard pemphigus treatment consists of oral corticosteroid, with/without adjuvant immunosuppressive drugs.^1^, ^8^ However, several morbidities associated with long-term daily corticosteroid use have been reported.^9^, ^10^

Rituximab was introduced for the pemphigus treatment in 2001.^11^ In the last years, it has been considered the first-line option to treat pemphigus in developed countries.^1^ Nevertheless, in Brazil, Rituximab has been prescribed as an exceptional option, for those cases of unresponsiveness to commoner drugs,^12^, ^13^, ^14^ which encouraged us to look for a diverse treatment in order to decrease corticoid side effects when chronically prescribed.^9^, ^10^

Knowing that autoimmune diseases are efficaciously controlled with pulse therapy regimens,^15^ besides the fact that we work in infirmaries for dermatological and rheumatological patients in shared clinical management, we introduced pulse therapy with Dexamethasone-Cyclophosphamide (DCP) or exclusive Dexamethasone (DP) in the treatment of pemphigus, based on Pasricha et al. (1998) first report.^16^

Here, we aimed to compare the outcomes of DCP/DP regimens in a Brazilian cohort of PV and PF patients according to the demographic and clinical data profiles.

## Materials and methods

### Type of study

Retrospective analytical study with medical records analysis in the last decade, in accordance with the STROBE checklist.

### Study population

Patients were assisted at the University Hospital, Ribeirão Preto Medical School, University of São Paulo, Brazil. Histopathology, direct and/or indirect immunofluorescence features plus ELISA with anti-desmogleins 1 and 3 commercial kits (MBL, Nagoya, Japan) confirmed pemphigus diagnosis and helped to allocate patients to the PV and PF groups. Inclusion criteria consisted of a confirmed diagnosis of PV or PF, and a unique cycle of DCP/DP therapy prescribed (see pulse therapy). Exclusion criteria were pregnancy, lactation, and previous treatment with Rituximab. Patients who had previous treatments were shifted to the pulse therapy regimen due to refractoriness and side effects of chronic daily oral prednisone, anti-inflammatory and/or immunosuppressive drugs (Table 1).

**Table 1:** Demographic and clinical data of pemphigus vulgaris (PV) and pemphigus foliaceus (PF) patients in DCP or DP pulse therapy regimens

	PV	PF	p-value
	n = 37	n = 41	
**Gender,** n (%)			0.819
Male	14 (37.8)	17 (41.5)	
Female	23 (62.2)	24 (58.5)	
**Age (years) median (min‒max)**	42 (16‒69)	21 (11‒85)	<0.001
**Patients under 18 years,** n (%)	2 (5.4)	10 (24.4)	0.028
**Disease duration before pulse therapy (months) median (min‒max)**	14 (2‒92)	69 (2‒528)	<0.001
**Pulse therapy as first treatment,** n (%)	5 (13.6)	None	0.055
DCP	4 (10.8)	None	
DP	1 (2.7)	None	
**Previous treatment,** n (%)^a^	32 (86.5)	41 (100)	
Prednisone	31 (83.8)	40 (97.6)	
Dapsone	15 (40.5)	12 (29.3)	
Hydroxychloroquine	1 (2.7)	14 (34.1)	
Azathioprine	7 (18.9)	9 (22.0)	
Methotrexate	1 (2.7)	8 (19.5)	
**Pulse therapy regimen** n (%)			0.235
DCP	27 (73.0)	24 (58.5)	
DP	10 (27.0)	17 (41.5)	

a Patients were treated with more than one drug.

### Pulse therapy

Pulse therapy regimens followed previous recommendations comprising four Phases.^17^ When Dexamethasone (DXM) and Cyclophosphamide (CYP) infusions are prescribed is named DCP therapy, and the regimen involving exclusive DXM infusion is designated DP therapy. DCP comprises 100 mg of DXM dissolved in 500 mL of 5% glucose and is given as an intravenous infusion and repeated on three consecutive days. On the second day, the patients also receive an infusion of CYP 500 mg. The young patients and patients who wish to have children are treated with DP, infusion of exclusive DXM 100 mg in three consecutive days.

Phase I comprises pulses every 28‒30 days until the patient achieves remission. If the patient does not achieve remission in Phase I, failure is considered. Phase II consists of nine consecutive pulses every 28‒30 days, and daily oral CYP 50 mg is maintained to consolidate the remission achievement. Phase III comprises exclusive daily oral CYP 50 mg for nine months. Phase IV consists of remission without medication, considered a post-treatment follow-up.

For DCP, the dose regimen of prednisone prescribed previously was maintained in Phase I. When remission occurs, the prednisone tapering dose was managed in Phase II. For DP, prednisone dose was managed in Phases I‒III.

We have a protocol of clinical and laboratory exams to elect the patient for pulse therapy, including teeth examination, and exams to discharge cancer. Before each pulse is prescribed in infirmaries, clinical and laboratory general exams are realized, mainly hematological, liver function and urine.

Remission consisted of the resolution of old lesions, and no more appearance of new lesions in Phase I. During Phases II and III, patients should remain free of new bullous lesions. If new blistering lesions are recurring, Phase I has to be restarted. When new lesions recur in Phases II‒IV, failure is considered. Abandonment was considered when the patient did not return in three months. Follow-up consisted of at least twelve months of weekly medical appointments with patients who reached Phase IV.

### Statistical analysis

Discrete and continuous data were expressed as count (percentage) and median (range), respectively. Mann-Whitney test was used for continuous variables as appropriate. Categorical variables were contrasted by using the Fisher-exact test. Statistical analysis was performed with Prism GraphPad 9 software (La Jolla, CA, USA). The significance level was set at α > 5%.

### Ethics

Patients signed the informed consent form and patients under 18 years had their consent form signed by their parents or legal guardians. The study was approved by the local Ethics Committee (#10-51729-2 and #8063-2015) in accordance with the ethical standards of the Helsinki Declaration of the World Medical Association.

## Results

The study included 78 pemphigus patients, 37 with PV and 41 with PF. Information about gender, age, disease duration and previous treatments before pulse therapy prescription, and allocation in DCP and DP regimens are described in Table 1. The two groups presented statistical differences in terms of age and duration of disease, being PV patients older than PF, and PF duration disease greater than PV (p < 0.001 for both). The allocation of PV and PF patients in DCP or DP regimens was similar (p = 0.235). Therefore, the results were analyzed comprehending both regimens, named DCP/DP therapy.

Five PV patients were allocated to the pulse regimen as a first option because of the severity of the disease.

Data on pulse therapy outcomes in Phases I‒IV for PV and PF groups are expressed in Table 2.

**Table 2:** Pulse therapy outcomes according to the Phase of DCP/DP regimens in pemphigus vulgaris (PV) and pemphigus foliaceus (PF) groups

	PV (n = 37)	PF (n = 41)	p-value
	n (%)	n (%)	
**Phase I**	37 (100)	41 (100)	
Remission, followed to Phase II	25 (67.6)	23 (56.1)	0.355
Failure	5 (13.5)	6 (14.6)	1.0
Abandonment	4 (10.8)	5 (12.2)	1.0
Clinical intercurrences	2 (5.4)	4 (9.7)	0.678
Continuing therapy^a^	1 (2.7)	3 (7.3)	N.A.
Pulses to remission, median (min–max)	6 (1–18)	10 (2–33)	0.005
Months to remission, median (min‒max)	5 (1‒19)	11 ( 2‒3 3)	0.002
Pulses to failure, median (min–max)	13 (11–24)	25 (16–34)	0.074
**Phase II**	25 (100)	23 (100)	
Maintained remission, follow to Phase III	21 (84.0)	19 (82.6)	1.0
Relapse	1 (4.0)	None	1.0
Abandonment	1 (4.0)	None	1.0
Clinical intercurrences	1 (4.0)	1 (4.4)	1.0
Continuing therapy^a^	1 (4.0)	3 (13.0)	N.A.
**Phase III**	21 (100)	19 (100)	
Maintained remission, follow to Phase IV	18 (85.7)	19 (100)	1.0
Abandonment	2 (9.5)	None	0.493
Clinical intercurrences	None	None	1.0
Relapse	1 (4.8)	1 (5.3)	1.0
Continuing therapy^a^	1 (4.8)	None	N.A.
**Phase IV**	17 (100)	18 (100)	
Remission off-therapy	14 (82.4)	14 (77.8)	1.0
Relapse	3 (17.6)	4 (22.2)	1.0
Time to relapse (months), median (min–max)	3 (2–3)	3 (1–47)	0.971
Follow-up duration (months), median (min–max)	12 (8–44)	26 (8‒56)	0.07

a Patients who still were in Phases I, II or III at data collection time; N.A., Not Applicable.

### DCP/DP outcomes in 37 PV patients

Of 37 PV patients, 25 (67.6%) acquired remission in Phase I after a median of 6 (1–18) pulses, and followed to Phase II. Failure (13.5%) was determined after a median of 13 (11–24) pulses, being those patients allocated in other therapy modalities, such as Rituximab. Two (5.4%) patients presented clinical intercurrences: one received treatment for latent tuberculosis and the other presented kidney cancer. In fact, the diagnosis of the patient who died of kidney cancer was revised, he presented paraneoplastic pemphigus. Twenty-one (84%) patients of Phase II followed to Phase III. In Phase II, 1 (4%) patient relapsed, and another (4%) presented suicidal ideation, as clinical intercurrence attributed to corticoids. In Phase III, 2 (9.5%) abandoned the treatment, and 1 (4.8%) presented relapse. Of 17 patients in Phase IV, 14 (82.4%) maintained remission off-therapy with a median follow-up of 12 (8–44) months, and 3 patients (17.6%) relapsed in 2‒3 months.

DCP was prescribed as the first treatment for 4 PV patients related to the severity of the disease (Table 1): 2 patients presented failure in Phase I and Rituximab was prescribed; one was in Phase I, and another one was in Phase IV. DP was prescribed as the first treatment for one PV patient, who relapsed after two months in Phase IV.

Table 3 shows a comparison of demographic and clinical data of 14 PV patients in remission off-therapy and 5 patients who presented failure in Phase I. Youngest PV patients presented greater frequency of failure (p = 0.031); the number of pulses and the duration of Phase I were smaller in patients who presented remission (p = 0.002 for both).

**Table 3:** Remission following Phase IV off-therapy or failure in Phase I outcomes of pulse therapy in pemphigus vulgaris and pemphigus foliaceus groups. Comparison of demographic, clinical data and the number of pulses and duration of Phase I

	Pemphigus vulgaris			Pemphigus foliaceus		
	Remission off-therapy	Failure in Phase I	p-value	Remission off-therapy	Failure in Phase I	p-value
	n = 14	n = 5		n = 14	n = 6	
**Gender** (n)			0.631			0.161
**Male**	4	2		4	4	
**Female**	10	3		10	2	
Median (min–max)						
**Age** (years)	50.5 (18–61)	36 (16–48)	0.031	23 (11–45)	36 (13–61)	0.247
**Disease duration before pulse therapy** (months)	14 (3–45)	11 (3–34)	0.374	81 (8-–528)	114 (5–162)	0.999
**Number of pulses in Phase I**	6.5 (2–18)	13 (11–24)	0.002	10.5 (3–33)	25 (16–34)	0.004
**Phase I duration** (months)	5.5 (2–19)	19 (10–33)	0.002	12 (4–33)	29.5 (22–51)	0.003

### DCP/DP outcomes in 41 PF patients

Of 41 PF patients, 23 (56.1%) acquired remission in Phase I after a median of 10 (2‒33) pulses and followed to Phase II. Failure was determined in 6 (14.6%) patients, after a median of 25 (16‒34) pulses. Five (12.2%) patients abandoned the treatment, and 4 (9.7%) presented clinical intercurrences (septic arthritis and skin infections) and were withdrawn from the study. Nineteen (82.6%) patients followed Phase III, 1 (4.4%) patient was withdrawn due to dental treatment, and another 1 (5.3%) relapsed. Of 18 patients in Phase IV, 14 (77.8%) maintained remission off-therapy with a median follow-up of 26 (8‒56) months. Meanwhile, relapsed happened in 4 (22.2%) after a median of 3 (1–47) months.

The clinical data comparing 14 PF patients who achieved remission off-therapy and 6 patients who presented failure in Phase I was shown in Table 3. The variables that statistically differed the outcomes were: the number of pulses and the duration of Phase I were smaller in patients who presented remission (p = 0.004 and p = 0.003, respectively).

### Comparison of DCP/DP outcomes in PV and PF patients who achieved remission off-therapy (Table 4)

DCP/DP outcomes were similar in both groups. In summary, 14 (37.8%) of 37 PV, and 14 (34.1%) of 41 PF were in remission, considered free of disease in Phase IV (p = 0.815). The failure occurred in 5 (13.5%) of 37 PV, and in 6 (14.6%) of 41 PF in Phase I (p = 1.0). Relapse in Phases II to IV occurred in 5 (13.5%) of 37 PF and in 5 (12.2%) of 41 PF (p = 1.0). Youngest PV patients presented a greater frequency of failure. PF patients were the youngest, presented a longer duration of disease, and need a higher number of pulses to achieve remission in Phase I.

**Table 4:** Comparison of demographic, clinical data and number of pulses and duration of Phase I between pemphigus vulgaris (PV) and pemphigus foliaceus (PF) groups that achieved remission off-therapy

	PV	PF	p-value
	n = 14	n = 14	
**Gender (n)**			1.00
**Male**	4	4	
**Female**	10	10	
Median (min–max)			
**Age** (years)	50.5 (18–61)	23 (11–45)	<0.001
**Disease duration before pulse therapy** (months)	14 (3–45)	81 (8–528)	0.006
**Number of pulses in phase I**	6.5 (2–18)	10.5 (3–33)	0.026
**Phase I duration** (months)	5.5 (2–19)	12 (4–33)	0.015
**Follow-up duration in phase IV** (months)	12 (8–44)	26 (8–56)	0.070

### Dropouts of pulse therapy

In general, pulse therapy was well tolerated, but minor side effects (skin and urinary infection, nausea and vomiting, headache, and hematuria) occurred in 3 (8.1%) of 37 PV, and in 5 (12.2%) of 41 PF (p = 0.715). We recorded a case of prostatic adenocarcinoma, diagnosed seven months after pulse therapy was suspended. No patient died due to treatment. Abandonment occurred in 7 (18.9%) of 37 PV, and in 5 (12.2%) of 41 PF groups (p = 0.534). In total, dropout was observed in 10 (27%) of 37 PV, and in 10 (24.4%) of 41 PF patients (p = 0.802).

## Discussion

Over the last decades, pulse therapy has become a safe option to treat pemphigus: it avoids the long-term side effects of oral daily treatment with corticosteroids and/or immunosuppressant drugs.^9^, ^10^, ^16^, ^17^, ^18^, ^19^, ^20^, ^21^ The main reported modalities of pulse therapy comprehend DCP and dexamethasone pulse with oral azathioprine, resulting in similar control of the disease, but a higher frequency of relapse in azathioprine regimen.^22^ In Brazil, a unique report on pulse therapy for pemphigus was achieved from the literature.^23^

DCP has been prescribed to more than 300 pemphigus patients in India; 190 (84%) of 227 patients who completed the treatment were free of disease (25.3% with follow-up of more than 5 years).^18^ The comparison of steroid-sparing adjuvants has shown DCP and cyclosporine the best drugs in terms of relapse.^19^

In the present study, we compared the outcomes of DCP/DP in the two main clinical forms of pemphigus in a Brazilian prevalent region for PV and PF.^3^ Considering the total sample of PV and PF groups, who completed one cycle of DCP/DP, the frequencies of remission were similar for both (14 [37.8%] of 37 PV, and 14 [34.1%] of 41 PF). If we consider the patients who followed to Phase IV, 14 (82.4%) of 17 PV and 14 (77.8%) of 18 PF presented remission with a median of follow-up of 12-months and 26-months, respectively.

The duration of Phase I determines the duration of complete pulse therapy. The factors that influence the Phase I are important to establish the prognosis of DCP/DP response. In Phase I, 25 (67.7%) of 37 PV, and 23 (56.1%) of PF acquired similar frequencies of remission. Noteworthy, while PF is considered to have a better prognosis than PV, the PF group has taken longer to acquire remission when compared to PV, with a median of 10 and 6 pulses (p *=* 0.005), and 11 and 5-months, respectively (p = 0.002). Roga, Augustine (2018) described 10-months for PV and 8-months for PF, but PF took more cycles than PV to achieve control.^22^ Mundakkat, Sridharan (2018) associated the longer duration of the induction phase (Phase I) with oral and skin score severity, and with the duration of illness.^24^ It is important to point out that DCP/DP, with exception of 5 PF cases, was prescribed to patients who did not present a response to previous treatment.

In fact, the disease duration before pulse therapy prescription was longer in PF compared to PV (median = 69-months and 14 months, respectively; p < 0.001). The PF epidemiological and clinical behavior could explain this finding ‒ PF usually begins at an early age, courses with low severity, allowing for a long period without the use of any aggressive therapeutic agent.^3^ These facts may justify the great number of pulses to acquire remission in the PF group seen in Phase I. Although the severity of PV is greater than PF, PV patients started pulse therapy earlier following a shorter period of the disease compared to PF, which could justify the faster response and the smaller number of pulses that were necessary to achieve remission in Phase I.^22^, ^24^

The frequencies of failure in Phase I was similar for PV and PF (13.5% and 14.6%, respectively) (Table 2). The number of pulses considered for failure was greater for PF, compared to PV (median = 25 and 13, respectively). In Phases II and III, few patients relapsed (2 PV and 1 PF). In Phase IV, when the patients do not use any corticoid or immunosuppressor, relapse was seen in 3 (17.6%) of 17 PV and in 4 (22.2%) of 18 PF patients. Searching for factors that could be influencing the outcomes of remission off-therapy in Phase IV, and failure in Phase I (Table 3), the number of pulses in Phase I was greater in the failure subgroup in both PV and PF. In terms of age, the youngest PV patients presented more frequency of failure compared to those in remission off-therapy (median = 36-y-old and 50.5-y-old, respectively). Incomplete or irregular treatment and/or single-medication pulse therapy could lead to relapse or therapeutic failure. For Pasricha et al., successful pulse therapy depends on the strict protocol and rigorous patient follow-up.^18^ Youngest PV patients were treated with a DP regimen, which could justify the highest frequency of failure.

PV and PF patients did not differ in terms of abandonment or clinical intercurrences. Abandonment occurred in 7 (18.9%) of 37 PV, and in 5 (12.2%) of 41 PF. Clinical intercurrences were noted in 3 (8.1%) of 37 PV, and in 5 (12.2%) of 41 PF patients. Considering dropout off-treatment, 27% of PV and 24.4% of PF did not finalize the treatment. Mahajan et al. (2022) reported a 41.5% dropout rate in pemphigus patients treated with DCP.^21^ We were able to identify not only difficult transportation from other cities to the reference center and personal and family problems as the main reasons for dropout but also abandonment without registered justification in the medical charts. Maintaining patients’ adherence to pulse therapy is certainly a challenge for physicians working with this therapeutic regimen. For the patients ongoing this treatment modality, we started scheduling weekly appointments with them before the attendance, which could explain the best adherence to treatment.^25^

Finally, pulse therapy is not free of side effects, being asthenia, fatigue, myalgia, arthralgia, flushing, palpitations, insomnia, headache, anorexia, and nausea and vomiting the most commonly described (see Ref.^26^ to review pulse therapy modalities).^21^, ^26^ Most of the patients tolerated pulse therapy well and presented with minor side effects. Notwithstanding, there was one case of prostatic adenocarcinoma, diagnosed seven months after pulse therapy was suspended. This 60-year-old patient had used CYP for 4.5 years with a cumulative dose of 20 g. Serious side effects reported for this drug, like lymphoproliferative diseases, hemorrhagic cystitis, and bladder cancer may occur after 30‒100 g of CYP cumulative dose.^27^ Furthermore, there are no reports to date relating CYP and prostatic cancer. Hence, we cannot directly attribute this case of prostatic cancer to DCP. Anyway, physicians should monitor the emergence of this kind of event. Although deaths during pulse therapy have been described, in this study no death was registered.^26^

Before the corticosteroids era, pemphigus lethality could reach 70%. Following the introduction of corticosteroids and adjuvant therapy, it dropped to less than 10%.^28^ Since its introduction in 1982 by Pasricha et al., DCP has been revolutionizing the pemphigus treatment. This modality provides not only the control but also the remission of pemphigus, with less associated severe side effects.^16^, ^19^, ^21^, ^24^^,^^29^

In developing countries, while we do not have Rituximab as a first-line sparing agent for the pemphigus treatment, DCP/DP should be considered as a safe modality to treat pemphigus.^30^ Since the present study has shown an association of higher failure with disease duration before pulse therapy, we recommend starting the pulse therapy promptly in mild-severe PV and PF disease.

The present results provide a better understanding of pulse therapy and may be useful for further studies aiming to clarify the association of the genetic profile of pemphigus patients with the DCP/DP treatment.

## Conclusions

PV and PF groups presented similar frequencies of DCP/DP outcomes: remission, failure, relapse and drop-out. Compared to PV, PF patients required a higher number of pulses to achieve remission during Phase I, which could be related to a longer disease duration before pulse therapy prescription.

## Financial support

This study was partially funded by FAPESP (Fundação de Amparo à Pesquisa do Estado de São Paulo) (#2010-51729-2) and FAEPA (Fundação de Apoio ao Ensino, Assistência e Pesquisa).

## Authors’ contributions

Ludmilla Figueiredo Vale Fontenelle: Data collection, analysis, and interpretation; statistical analysis; writing of the manuscript; effective participation in the research guidance; critical review of the literature; final approval of the final version of the manuscript.

Roberto Bueno-Filho: Analysis and interpretation; critical review of important intellectual content; final approval of the final version of the manuscript.

Sebastián Vernal: Analysis and interpretation; critical review of important intellectual content; final approval of the final version of the manuscript.

Renata Delfino: Data collection, analysis, and interpretation; final approval of the final version of the manuscript.

Giovanna Stefanne Lópes Barbosa: Analysis and interpretation; critical review of important intellectual content; final approval of the final version of the manuscript.

Eduardo Antonio Donadi: Analysis and interpretation; critical review of important intellectual content; final approval of the final version of the manuscript.

Ana Maria Roselino: Study concept and design; critical review of important intellectual content; effective participation in the research guidance; critical review of the literature; final approval of the final version of the manuscript.

## Conflicts of interest

None declared.
